# Acute Retroviral Syndrome Presenting as Acute Hepatitis

**DOI:** 10.7759/cureus.3755

**Published:** 2018-12-20

**Authors:** Ahmad A Abu-Heija, Maya Shatta, Ahmed Yeddi, Anand Kumar Ravi, Milton Mutchnick

**Affiliations:** 1 Internal Medicine, Wayne State University, Detroit, USA; 2 Gastroenterology, Wayne State University, Detroit, USA

**Keywords:** acute hiv, acute retroviral syndrome, primary hiv, acute hepatitis, hepatitis, transaminitis, anti-retroviral treatment, hiv

## Abstract

Acute retroviral syndrome (ARS) can present as a wide array of clinical manifestations. Establishing a diagnosis early in the disease course can provide an opportunity to minimize immunosuppression and limit further transmission of human immunodeficiency virus (HIV). We present a case of a previously healthy young male who presented with acute hepatitis, as a manifestation of ARS. Initial HIV antigen/antibody testing was negative; however, a high index of suspicion prompted HIV ribonucleic acid (RNA) virologic testing revealing >10 million RNA copies/mL. Anti-retroviral treatment was initiated, along with supportive measures, accomplishing resolution of the transaminitis and the restoration of CD4 counts within normal at one month. Early in the disease course, HIV screening immunoassay could still be negative; hence, confirmatory testing with HIV RNA virologic testing should be pursued when clinical suspicion is high. Prompt diagnosis and treatment can improve outcome and curtail viral transmission.

## Introduction

The incidence of human immunodeficiency virus (HIV) in the United States has remained consistent over the past few years, with approximately 40,000 new cases diagnosed annually [1]. African Americans, males, and men who have sex with men (MSM) disproportionately constitute the majority of these newly diagnosed cases [1-2].

Acute retroviral syndrome (ARS) is a clinical syndrome entailing symptoms that present during the first three to six months of HIV infection [3]. This syndrome is also referred to as primary HIV infection (PHI) and ARS [2-3]. ARS was first described in 1985 by Cooper et al. in an article in The Lancet; this syndrome was described as a mononucleosis-like syndrome characterized by a constellation of symptoms including fever, sweats, malaise, lethargy, anorexia, and lymphadenopathy [4]. Subsequent studies have shown that ARS can present with an assortment of pathological manifestations to a multitude of bodily systems, including, but not limited to, neurological, gastrointestinal, dermatological, respiratory, and urological systems [3,5-8]. Patients who seek medical attention with ARS constitute around 85% of the total number of patients; yet a disproportionate number of these patients face a delay in diagnosis, given the non-specific presentation of ARS and array of manifestations on evaluation [3,7].

## Case presentation

We present a 25-year-old patient with a remote medical history of successfully treated primary syphilis, who presented to our hospital chiefly complaining of back pain, headache, diarrhea, and vomiting over the course of the preceding two days. The patient reported feeling ill for three days prior to presentation and was prompted to visit the emergency department after his acute illness failed to improve with ibuprofen. On examination, the patient was febrile with a temperature of 38.5 °C (101.3 °F) and tachycardic with a heart rate of 123. The patient was normotensive with a blood pressure of 130/78 mmHg and in a moderate degree of distress. Neurological examination revealed normal muscular strength in both bilateral lower and upper extremities, in addition to an absence of sensory deficits. Abdominal examination was normal, with no evidence of organomegaly or tenderness to abdominal palpation. Examination of his precordium and lungs was normal.

Biochemical and hematological investigations revealed a low-normal leukocyte count of 3,900 cells/µL (normal 3,500 to 10,600), elevated hemoglobin of 17.6 gm/dL (normal, 13.3 to 17.1), an elevation in creatinine levels of 1.76 mg/dL (normal, 0.70 to 1.30), elevated aspartate transaminase (AST) levels of 49 U/L (normal <39), normal alanine transaminase (ALT) levels of 30 U/L (normal <52), and normal total bilirubin levels of 0.31 mg/dL (normal <1.00). Blood cultures were obtained, and a single dose of vancomycin, cefepime, and metronidazole was administered. Further assessment of the patient revealed that he is in a monogamous relationship with an HIV-positive male partner and regularly participates in unprotected anal intercourse. The patient also revealed that he declined pre-exposure prophylaxis (PrEP) previously, as his partner consistently had an undetectable HIV viral load. Rapid HIV testing with a fourth-generation antigen-antibody test yielded a negative result. Antibiotics were discontinued, on the second day of admission, due to a low clinical suspicion for an underlying bacterial infection.

**Figure 1:**
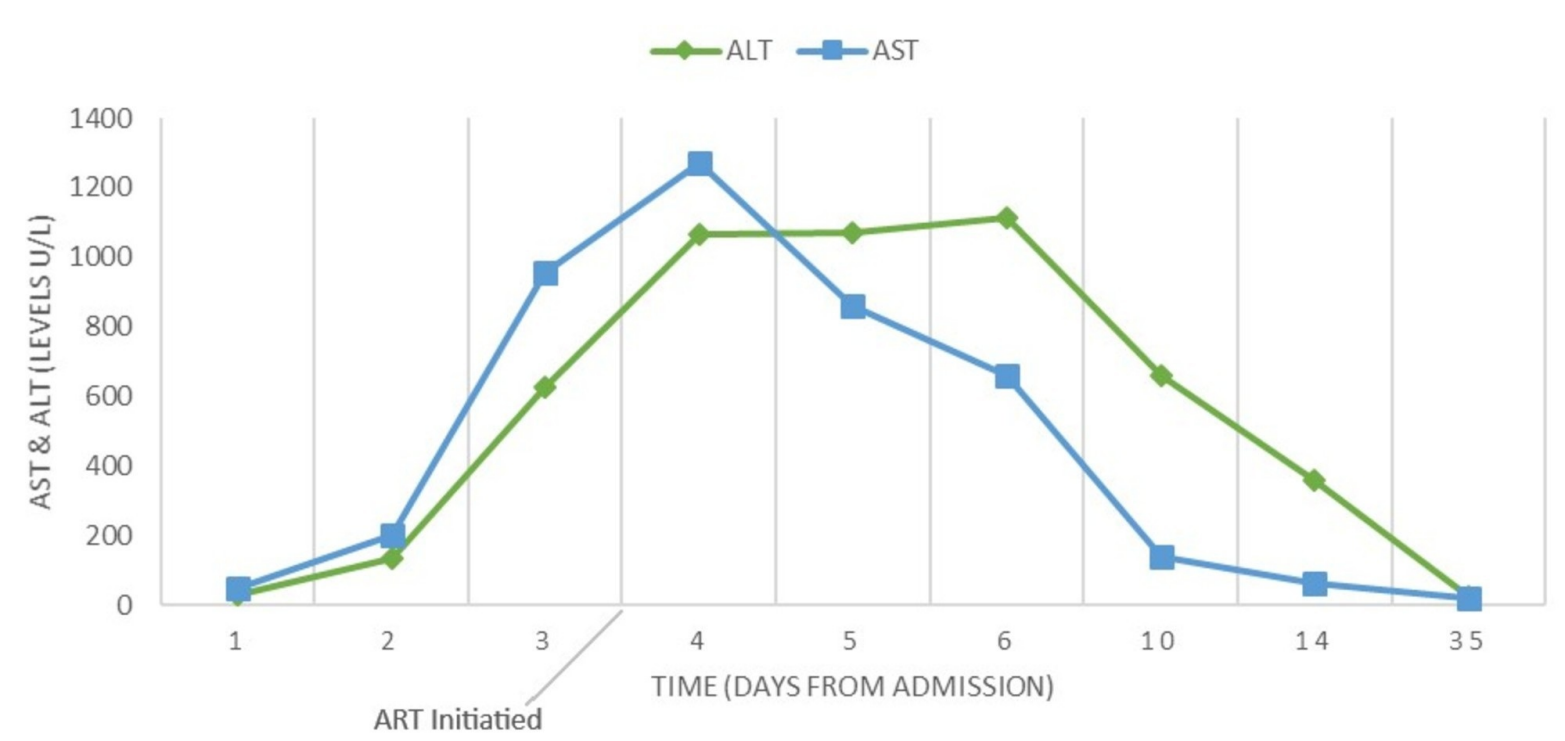
Line chart displaying the change in transaminase levels ART: anti-retroviral treatment

After admission, the patient continued to experience febrile episodes with a peak temperature on the third day of admission at 39.3 °C (102.7 °F), with the resolution of his diarrhea and back pain on the second day of admission. Repeat laboratory investigations on the second day revealed further elevation in AST and ALT levels to 200 U/L and 137 U/L, respectively, with a continued rise as observed in Figure 1 throughout the first five days of hospitalizations. Moreover, the patient developed evidence of coagulopathy with an elevation in the international normalized ratio (INR) to 1.45 (normal <1.00) and partial thromboplastin time (PTT) to 62.4 seconds (normal, 23.1 to 33.1). Total bilirubin and alkaline phosphatase levels remained normal throughout the hospitalization.

Investigations were undertaken to rule out an infective etiology for the transaminitis. Serological studies including immunoglobulin M to hepatitis A (HAV IgM), hepatitis B surface antigen (HBsAg), hepatitis B e antigen (HBeAg), immunoglobulin M to hepatitis B core antigen (HBc IgM), anti-hepatitis C virus antibody (anti-HCV), hepatitis C RNA by polymerase chain reaction (PCR), immunoglobulin M to hepatitis E (HEV IgM), polymerase chain reaction (PCR) for Herpes Simplex virus (HSV) 1 & 2 DNA, immunoglobulin M to Ebstein-Barr virus (EBV IgM) and cytomegalovirus (CMV IgM) were all negative. Liver ultrasound was normal. Serological testing was also performed to rule out other etiologies, including anti-nuclear antibody (ANA), anti-smooth-muscle antibody, anti-mitochondrial antibody, liver-kidney microsomal antibody (anti-LKM-1) which were all negative. Alpha-1 antitrypsin levels were normal at 159 mg/dL (normal, 90-200) and ceruloplasmin levels were normal at 226 mg/L (normal, 180-580), and ferritin levels were elevated at >7,500 ng/mL (normal, 24-336). Genotypic testing (C282Y) for hemochromatosis was negative.

Given our high index of suspicion for primary HIV, subsequent ultrasensitive testing for HIV viral load was performed using COBAS Ampliprep/COBAS TaqMan HIV-1 assay (TaqMan, Roche Diagnostics, Mannheim, Germany), which revealed >10,000,000 HIV-1 ribonucleic acid (RNA) copies/mL, confirming primary HIV infection, indicative of acute seroconversion. CD4 and CD8 cell counts were 689 and 506 cells/µL comprising 28% and 21% of lymphocytes, respectively. After an extensive discussion with the patient, the decision was made to start the patient on anti-retroviral treatment (ART) with emtricitabine, tenofovir alafenamide, and dolutegravir. After the fifth day of admission, there was a downward trend in the transaminase levels, and the patient was discharged with a follow-up with his primary care doctor and the HIV clinic. On follow-up, one week after discharge, laboratory investigations revealed progressive improvement in transaminases with resolution at one month, as seen in Figure 1. CD4 count at one-week follow-up was 268 cells/µL and improved to 648 cells/µL after one month of ART. Furthermore, at one month, the HIV viral load declined to 376 HIV-1 RNA copies/mL.

## Discussion

We present the case of a young man who engaged in sexual intercourse with an HIV-infected male partner and presented with acute hepatitis. Infectious etiologies that could be responsible for hepatitis were excluded, including hepatitis A, B, C, and E, CMV, HSV, and EBV. Additionally, structural and non-infectious etiologies were excluded, including autoimmune hepatitis, hemochromatosis, Wilson’s disease, and alpha-1 antitrypsin deficiency. His disease process was considered a manifestation of ARS. Discussions regarding obtaining a liver biopsy ensued in the initial workup of the disease process; however, given the resolution in liver enzyme elevation and clinical improvement, the decision was made to avoid an unnecessary invasive procedure.

Universally, HIV remains an epidemic with many yet unidentified infected persons. In the United States alone, an estimated 162,500 individuals are infected with HIV with a yet unconfirmed diagnosis [1]. A multitude of barriers hinder a timely diagnosis, and an atypical presentation exacerbates this discrepancy. As described by Braun et al., the median time between the estimated date of infection and the first positive antibody test among patients presenting to a hospital with ARS ranged between 20 and 61 days [3]. During the initial phase of ARS, there is an extremely elevated HIV RNA viral load without circulating the antibodies. Earlier identification of HIV infection yields manifold public health benefits, given early infection is often associated with very high viral loads correlating with a higher possibility of ongoing viral transmission., especially with continued high-risk behavior [9]. Furthermore, early ART initiation reduces the burden of immunosuppression, serious infections, and mortality and by virtue of reducing the HIV RNA levels, has the potential to curtail transmission of the infection [10].

Variability in the presentation is among the most important deterrents in establishing a diagnosis of ARS, as presentation varies widely ranging from a simple flu-like illness to catastrophic multi-organ failure [2,5-6]. Physician unfamiliarity with ARS and the investigations necessary to establish the diagnosis are other factors that preclude early identification and diagnosis. In a study by Weintrob et al., it was demonstrated that more than 50% of the patients diagnosed with ARS were evaluated in at least three separate healthcare encounters before ARS diagnosis was established, and only 17% were diagnosed with ARS during the initial encounter [11]. The majority of providers and laboratories utilize a combination screening immunoassay (combination antigen/antibody immunoassay) as an initial investigation when ARS is suspected. However, in the early HIV infection, this test could be negative and a high index of suspicion warrants the utility of HIV RNA testing with either qualitative or quantitative tests. Quantitative HIV RNA virologic testing is the preferred modality as it reports the actual number of copies of virus per milliliter of blood and in cases of ARS is the first test to become positive [2].

Moreover, emphasis should be placed during all encounters with patients who engage in high-risk behavior, including sexual intercourse with HIV-infected partners or IV drug use, on the initiation of PrEP with daily tenofovir and emtricitabine. Approximately 1.1 million people are at high risk of acquiring HIV in the United States, including a disproportionately high rate of black persons accounting for around 500,000 individuals, of whom only 7% are prescribed PrEP [12]. PrEP efficacy has been extensively studied, and variable efficacy rates were achieved consistently among the various studied populations, including MSM and IV drug users. Nevertheless, significant efficacy in reducing HIV transmission, approaching 90%, has been demonstrated with adequate adherence [13].

The precise underlying mechanism responsible for liver enzyme elevation in acute HIV infection is not clearly understood. However, molecular schemes have shown that HIV induces hepatocyte apoptosis, contributing to an elevation in liver enzymes [14]. Tumor necrosis factor (TNF)-related apoptosis is stimulated by HIV glycoproteins [15]. In addition, interleukin (IL)-8 appears to play an important role in hepatic inflammation, which is induced by Gp120 [14]. Studies on mice have shown improvement in hepatic synthetic function after ART initiation [16]. Furthermore, hepatocyte apoptosis is largely influenced by Kupffer cells, which appear to be activated by engulfed apoptotic hepatocytes, allowing for the development of liver inflammation [14].

## Conclusions

Establishing a diagnosis of ARS is clinically challenging. Nonetheless, physicians should remain vigilant while investigating the etiology of acute hepatitis. Prevalent infectious and non-infectious etiologies should be excluded in the initial evaluation of transaminitis. Other less common etiologies, including ARS, should be in the differential diagnosis of such a presentation in a patient with an increased risk of HIV infection. An initial negative HIV screening immunoassay should not preclude further confirmatory HIV RNA virologic testing in an appropriate clinical scenario. Prompt diagnosis and early linkage to care carry important implications to patients’ well-being and can improve outcomes while providing a venue for prevention of further HIV transmission.
